# Editorial: Tissue Engineering and Cell Therapy for Cartilage Restoration

**DOI:** 10.3389/fcell.2022.947588

**Published:** 2022-07-01

**Authors:** Tiago Lazzaretti Fernandes, Daniela Franco Bueno, Kazunori Shimomura, Zhenxing Shao, Andreas H. Gomoll

**Affiliations:** ^1^ Sports Medicine Division, Institute of Orthopedics and Traumatology, Hospital das Clínicas, Faculdade de Medicina, Universidade de São Paulo, São Paulo, Brazil; ^2^ Hospital Sírio-Libanês, São Paulo, Brazil; ^3^ Department of Orthopaedic Surgery, Osaka University Graduate School of Medicine, Suita, Japan; ^4^ Institute of Sports Medicine, Peking University Third Hospital, Beijing, China; ^5^ Department of Orthopaedic Surgery, Hospital for Special Surgery, New York, NY, United States

**Keywords:** tissue engineering, cell therapy, cartilage restoration, innovation, stem cells, cartilage, osteoarthritis

Articular cartilage is a connective tissue consisting of two phases: a solid composed of collagen, proteoglycans, proteins, and chondrocytes, and a liquid made up of water and electrolytes. It can be divided into four zones (superficial, middle, deep and calcified), each presenting a different cellular organization, collagen fiber architecture and depth (Hernandez et al., 2000; Meyers and Chawla, 2008; Sophia Fox et al., 2009). These structures and components establish the properties of cartilage, in terms of stiffness, elasticity and other important aspects for its characterization.

It is known that articular cartilage is an avascular tissue with little cellularity, making it difficult to heal. As a consequence, cartilage injuries can generate several complications for the individual, such as loss of mobility, degeneration and osteoarthritis (OA), directly affecting the quality of life. Not to mention that it represents an economic burden (Fernandes et al., 2018; Fernandes et al., 2020).

Current procedures that seek to repair chondral tissue are microfracture stimulation, autograft and osteochondral allograft, and cellular implant therapies based on tissue engineering principles. Cell therapies and regenerative techniques are important and there are two main examples: autologous chondrocyte implantation (ACI) and mesenchymal stromal cells (MSCs). MSCs have received considerable research attention, due to ease of collection, ability to proliferate and differentiate cells, non-rejection by the patient, and paracrine effect on local cellular machinery (Ando et al., 2007; Shimomura et al., 2015).

This Research Topic aimed to widening the knowledge on the strategies being used for articular cartilage regeneration, describing the state-of-the-art and exploring the innovative approaches in cartilage restoration field. This issue currently includes 14 articles related to different innovative propositions associated with cartilage injuries.

In Table 1, a summary and the innovative aspects of each article are presented.

**TABLE 1:** Innovative contribution of the articles published in Research Topic Tissue Engineering and Cell Therapy for Cartilage Restoration.

Authors	Title	Innovative aspect
Dai et al.	LncRNA H19 Regulates BMP2-Induced Hypertrophic Differentiation of Mesenchymal Stem Cells by Promoting Runx2 Phosphorylation	New mechanism to take into consideration when dealing with cartilage tissue engineering treatment. Regulation function of H19 in hypertrophic differentiation of cartilage
Hanai et al.	Potential of Soluble Decellularized Extracellular Matrix for Musculoskeletal Tissue Engineering – Comparison of Various Mesenchymal Tissues	New musculoskeletal therapy using decellularized ECM derived from mesenchymal tissues.
		Tissue derived soluble ECMs could be employed to regenerate tissues combined with some appropriate scaffolds seeded with some appropriate stem cells.
Wang et al.	BMSC-derived small extracellular vesicles induce cartilage reconstruction of temporomandibular joint osteoarthritis *via* autotaxin-YAP signaling axis	Novel treatment for Temporomandibular joint osteoarthritis (TMJOA) using BMSC-sEVs for cartilage reconstruction
Tejedor et al.	Pyrroline-5-Carboxylate Reductase 1 Directs the Cartilage Protective and Regenerative Potential of Murphy Roths Large Mouse Mesenchymal Stem Cells	The first evidence that MRL MSCs exhibit enhanced chondrogenic, chondroprotective, and regenerative properties as compared with BL6 MSCs in a Pycr1-dependent manner.
Tejedor et al.	MANF Produced by MRL Mouse-Derived Mesenchymal Stem Cells Is Pro-regenerative and Protects From Osteoarthritis	New therapy using MSC derived from MRL mouse as a cartilage regenerative potential, through their high capacity to release MANF.
Da Cruz et al.	Post-adipose-derived stem cells (ADSC) stimulated by collagen type V (Col V) mitigate the progression of osteoarthritic rabbit articular cartilage	Novel OA treatment: post-ADSC stimulation by Col V treatment increased the repair process in an osteoarthritic rabbit articular cartilage model
Yang et al.	Biofunctionalized structure and ingredient mimicking scaffolds achieving recruitment and chondrogenesis for staged cartilage regeneration	Novel strategy for articular cartilage regeneration: endogenous cell recruitment: growth factor-β3 (TGF-β3)-loaded biomimetic natural scaffold based on demineralized cancellous bone (DCB) and acellular cartilage extracellular matrix (ECM). Great promise for clinically effective *in situ* articular cartilage regeneration.
Li et al.	Autologous Fractionated Adipose Tissue as a Natural Biomaterial and Novel One-Step Stem Cell Therapy for Repairing Articular Cartilage Defects	Novel biomaterial, autologous fractionated adipose tissue (ECM/SVF-gel) to facilitate cartilage injury repair. It’s simple, time-sparing, cost-effective, minimally invasive, and enzyme-free preparation process
Mustapich et al.	A Novel Strategy to Enhance Microfracture Treatment With Stromal Cell-Derived Factor-1 in a Rat Model	New strategy that can enhance microfracture procedures and significantly improve the quality of cartilage regeneration in an osteochondral defect.
		Incorporating SDF-1 into an implantable scaffold yielded a superior quality of cartilage repair when compared with microfracture alone.
Lu et al.	Unfavorable Contribution of a Tissue-Engineering Cartilage Graft to Osteochondral Defect Repair in Young Rabbits	New model for studying treatment of cartilage defects. dECM-based tissue-engineering approach is able to repair adult cartilage defects, but is different for premature tissue.
Jacob et al.	Osteochondral Injury, Management and Tissue Engineering Approaches	Literature review combination on the assessment of different techniques for osteochondral lesions
Li et al.	Cell Interplay in Osteoarthritis	Summary of key cells that might be targets of future therapies for OA.
Najar et al.	Mesenchymal Stromal Cell Immunology for Efficient and Safe Treatment of Osteoarthritis	New strategy involving immunological features for developing MSCs as a therapeutic option for OA with high quality, safety and efficiency standards.
Pachito et al.	Technical Procedures for Preparation and Administration of Platelet-Rich Plasma and Related Products: A Scoping Review	Scoping Review: Combined studies of different methods for each stage of PRP processing, that can be used in different clinical situations of Medicine and Dentistry

Stem-cell-based and gene-enhanced tissue engineered cartilage is promising in the treatment of cartilaginous pathologies, especially traumatic cartilage defects (Bishop et al., 2017; Pirraco et al., 2018; Wang et al., 2019). Bone morphogenetic protein 2 (BMP2) triggers hypertrophic differentiation after chondrogenic differentiation of MSCs, which blocked the further application of BMP2-mediated cartilage tissue engineering. Dai et al. investigated the function of LncRNA H19 (H19) in BMP2, finding out that H19 regulates BMP2-mediated hypertrophic differentiation of MSCs by promoting the phosphorylation of Runx2, that maturates chondrocytes. H19 may play a role in cartilage differentiation, cartilage phenotype maintaining, and cartilage hypertrophic differentiation. They evaluated nine types of porcine tissue through a simple standard decellularization protocol.

At present, tissue engineering and regenerative medicine have focused on extracellular matrices (ECMs) to function as a natural scaffold (Harrison et al., 2014). Hanai et al. found that a soluble decellularized ECM (dECM) for each of the nine tissues harvested exhibited variations in their biochemical characteristics and growth factor distribution. On cell culture, it appeared to promote cell differentiation toward the specified used ECM tissue phenotype and decellularization was successful with reducing cellular components in every tissue. The present results are important in the field of musculoskeletal regeneration therapy, since tissue derived soluble ECMs could be employed to regenerate tissues combined with some appropriate scaffolds seeded with stem cells.

Researching less invasive treatments for Temporomandibular joint osteoarthritis (TMJOA), Wang et al. attempted to analyze the cartilage reconstruction effect of bone marrow MSC-derived small extracellular vesicles (BMSC-sEVs) and showed an increase of the tissue in the cartilage lacuna and hypertrophic cartilage cells in the deep area of the bone under the cartilage, besides higher rates of cell proliferation and migratory activity and alleviated G1 stagnation of the cell cycle of OAsEV which may provide guidance regarding their therapeutic applications as early and minimally invasive therapies for TMJOA.

Comparing Murphy Roths Large (MRL), who possess remarkable capacity to regenerate several musculoskeletal tissues, and BL6 mices regeneration to a ear punch, Tejedor et al. demonstrated that the enhanced regenerative potential of MRL mice is attributed, in part, to their MSCs that exhibit PYCR1-dependent higher glycolytic potential, differentiation capacities, chondroprotective abilities, and regenerative properties than BL6 MSCs, what could be a promising tool in the treatment of OA. Comparing the MSCs of these two mouse species, Tejedor et al. showed that the slow-proliferating MRL MSCs display a higher migration potential than the fast-proliferating BL6 MSC and found that mesencephalic astrocyte derived neurotrophic factor (MANF) was specifically highly produced by MRL MSC as compared to MSC derived from other mouse strains, and that MANF highly produced by MRL MSC contributes to their capacity to tend to reduce the OA score.

To delay the development of OA and promote joint cartilage regeneration, Da Cruz et al. demonstrated that post-adipose-derived stem cells (ADSC) stimulation by type V collagen (Col V) treatment induced a significant regeneration of cartilage in an osteoarthritic rabbit articular cartilage model suggesting that surgical-induced OA treated with ADSCs stimulated by Col V may prevent the progression of cartilage injury and indicating that ADSCs/Col V may be a therapeutic target for the treatment of osteoarthritis. Also using a defect produced in the articular cartilage of rabbits, Yang et al. developed a staged regeneration strategy that combines endogenous cell recruitment and pro-chondrogenesis approaches for *in situ* articular cartilage regeneration with growth factor (GF)-loaded scaffold who facilitated cell homing, migration, and chondrogenic differentiation and promoted the reconstructive effects of *in vivo* cartilage formation.

Studying the regeneration of defects in articular cartilage, Li et al. used extracellular matrix/stromal vascular fraction gel (ECM/SVF-gel) to evaluate the therapeutic effect of this natural biomaterial on this repair, comparing the control group treated with microfractures to the group with microfractures associated with ECM/SVF-gel, finding that autologous ECM/SVF-gel displays a curative effect on articular cartilage regeneration in a rabbit model, besides being a simple, time-sparing, cost-effective, enzyme-free and minimally invasive preparation process.

Microfracture is one of the most widely used techniques for the repair of articular cartilage. However, microfracture often results in filling of the chondral defect with fibrocartilage, which exhibits poor durability and sub-optimal mechanical properties. Mustapich et al. studied a strategy for enhanced microfracture treatment with stromal cell-derived factor-1 (SDF-1), demonstrating a simple cost-effective one-step process for improving the quality of cartilage defect repair in a rat model of microfracture. In this perspective, Lu et al. found that this approach played a unique role in cartilage resurfacing of adult rabbits despite the fact that self-healing dominates cartilage repair in young rabbits less than 9 months old.

After categorizing treatment options for osteochondral lesions into repair and regenerative techniques, Jacob et al. showed emerging techniques, exploring tissue engineering with new methods of cultures and their results, and stated that for improved chondrogenesis the cells require both growth factors and mechanical forces to bring about more physiological cellular responses. The author demonstrated satisfactory results in the medium-term follow-up with the MaioRegen presenting faster return to sports and a low rate of complication and failure, while the Agili-C showed encouraging results through significant improvement in the assessments.

OA is a multifactorial disease that presupposes local and systemic factors and has multiple pathogenetic mechanisms, which must be considered when exploring new treatment options. Li et al. presented the cells in the joint tissues and their role in OA pathogenesis, the numerous ways these cells communicate, and concluded that application of stem cell-derived EVs in OA treatment is an emerging field in regenerative medicine.

Cartilage tissue engineering has potential for the treatment of cartilage pathologies, providing biomaterials that can be adjuvants to already established treatments to improve the clinical outcome, providing a higher quality regeneration and in the shortest possible time. There are clearly several cellular regulatory pathways involved in the therapeutic effect of MSCs, and the broad cellular and molecular changes that accompany MSC apoptosis, autophagy, and senescence may be essential for their therapeutic effects according to Najar et al. This is an essential understanding of the immunological profile and functions of MSCs as a graft and understanding of the mechanisms involved in the effects of MSCs for better therapeutic targeting.

A treatment widely used in different clinical situations is Platelet-rich plasma (PRP), but universal standardization of procedures for its preparation is still lacking. Pachito et al. reviewed thirty-nine studies focusing on the comparison of PRP to a related product, types of anticoagulants, centrifugation protocols, commercial kits, processing time, methods for activation, and application concomitantly to other substances, finding a great variability embed in each step necessary for the preparation of PRP which may justify the variability of clinical effects of PRP across different clinical trials.

## Final Considerations

In this editorial, it was possible to discover a large number of innovative aspects, with extremely promising research fields that could change the course of the treatment of OA and other pathologies that affect the articular cartilage. Despite that, the articles have some methodological limitations and further research should be carried out focusing on the difficulties raised, in order to increase the level of evidence, safety and efficacy of the new therapies.

Innovative technologies and discoveries take time to be translated from an idea to clinical practice (Fernandes et al., 2022). Moreover the continuing development of the mentioned studies is of key importance to create collaborative learning and reach research goals for cartilage treatment and, at the end, improve healthcare and patient’s quality of life.
